# Rebound Exercises in Rehabilitation: A Scoping Review

**DOI:** 10.7759/cureus.63711

**Published:** 2024-07-02

**Authors:** Manisha A Rathi, Reema Joshi, Pinal Munot, Sakshi Pandit, Chaitanya A Kulkarni

**Affiliations:** 1 Physiotherapy, Community Based Rehabilitation, Dr. D. Y. Patil College of Physiotherapy, Pune, IND; 2 Physical Medicine and Rehabilitation, Community Based Rehabilitation, Dr. D. Y. Patil College of Physiotherapy, Pune, IND; 3 Public Health, Datta Meghe Institute of Higher Education and Research, Wardha, IND; 4 Community Health Physiotherapy, Ravi Nair Physiotherapy College, Wardha, IND

**Keywords:** scoping review, exercise, rehabilitation, physical fitness, mini trampoline, rebounding

## Abstract

The term "trampoline" was coined in 1969, introducing a dynamic feedback mechanism for exercise. Rebounding exercise on a mini-trampoline utilizes an elastic surface supported by springs and gravity, potentially reducing cumulative trauma from repetitive loading. This type of physical activity provides enjoyable and engaging exercise for adolescents, especially those who are overweight, thereby reducing the likelihood of injuries associated with exercise. Mini-trampoline exercises enhance blood circulation, oxygen delivery, and bone health, impacting lower limb strength, balance, motor performance, blood glucose levels, executive function, physiological markers, and overall quality of life. The study focused on examining the overall impact of rebounding exercises in the field of rehabilitation. Its main goal was to assess how these exercises affect the rehabilitation process and different health measures. By investigating the comprehensive influence of rebounding exercises, the study aimed to determine their effectiveness in aiding physical and functional recovery, targeting specific rehabilitation goals, and enhancing overall health outcomes. We systematically reviewed medical literature databases such as PubMed, MEDLINE, Scopus, Google Scholar, and EBSCO. We included research articles, systematic reviews, meta-analyses, clinical trials, case studies, and observational studies published in English up to 10 years before the review's cutoff in December 2023. We considered participants across all age groups. Articles not in English were excluded from the review. The outcome measures were body composition, waist-hip ratio, Bruininks-Oseretsky test for motor proficiency, reaction time, insulin resistance, lipid profile, blood cholesterol level, forced expiratory volume in one second, and forced vital capacity, bone health indicators, blood lactate level, balance, strength: repetitive maximum, brief pain inventory (short form). A total of 11 reports met these criteria. In conclusion, this review provides a thorough look into the use, challenges, and future potential of rebound exercises in rehabilitation and fitness. Despite their wide-ranging applications, issues such as insufficient research, equipment variability, and safety concerns persist. Advancement requires more research for evidence-based guidelines, improved equipment design and safety measures, and collaboration among researchers, clinicians, and manufacturers. Overcoming challenges and fostering innovation can establish rebound exercises as a valuable tool in rehabilitation and fitness.

## Introduction and background

In 1936, the term trampoline was initially coined, offering individuals the chance to engage with more exciting stimuli compared to mere dynamic feedback. Rebounding exercise involves utilizing a mini-trampoline to facilitate vertical movement of the body, leveraging the bouncing effect of the mini-trampoline [1]. Trampoline-based exercise utilizes an elastic surface supported by springs and gravity, potentially reducing the frequency of cumulative trauma resulting from repetitive loading due to the activity's minimal jarring effect [2]. Engaging in rebounding on a mini-trampoline offers children a pleasurable and stimulating exercise experience, potentially masking the intensity of their physical exertion [3]. In 2018, a study found that adolescents prefer physically engaging and enjoyable activities that are cost-effective, offer sociability, and provide appropriate facilities. As time progresses, maintaining regular exercise can become challenging for many adolescents. Given that overweight and obese individuals are at higher risk of exercise-related injuries due to their excess body weight, utilizing a compliant mini-trampoline for aerobic exercise appears to present an advantageous alternative to traditional forms of exercise such as jogging, treadmill use, running, skipping, or dancing [4]. This contemporary apparatus offers considerable advantages to the human body by facilitating both rhythmic and nonrhythmic floor exercises. Its rubberized pad mitigates joint stress during physical activities, thereby enhancing blood circulation and oxygen delivery to the body's cells. This heightened oxygenation boosts individual energy levels and positively impacts bone health. This review thoroughly examines the multifaceted aspects of mini-trampoline exercises and their importance in rehabilitation, with a focus on implications such as enhancing lower limb strength [2], balance training [5,6], motor performance, blood glucose level [7], executive function [8], physiological markers [9], body stabilization, muscle coordinative responses, physical fitness, joint movement amplitudes, and spatial orientation. Additionally, it offers benefits for managing body weight, controlling blood glucose levels, and enhancing overall quality of life, thereby warranting inclusion in obesity management strategies.

## Review

Methodology

The literature search strategy was carried out, and a comprehensive review was conducted to gather pertinent studies on utilizing mini-trampoline exercises in rehabilitation. Various electronic databases, such as PubMed, Medline, Scopus, Google Scholar, and Ebsco, were systematically searched using a combination of keywords and Medical Subject Headings terms, including "Mini Trampoline" and "Rebound Exercises." Boolean operators (AND, OR) were employed to enhance the precision of the search outcomes. The inclusion criteria were medical literature, including research articles, systematic reviews, meta-analyses, clinical trials, case studies, and observational studies, published in English up to a decade before the review's cutoff date in December 2023. Participants of all age groups were considered. Articles written in languages other than English were excluded from the review. The study selection was done with the titles and abstracts of identified articles, which were assessed by two impartial reviewers to ascertain their relevance and eligibility. In cases of disagreement, input from a third reviewer was sought to achieve consensus. Selected studies underwent a comprehensive full-text review with documented exclusion criteria. Data gleaned from a few studies were extracted using a systematized data extraction form. The following information was collected from the articles: title, authors, and publication year. Study design and methodology include population characteristics (e.g., age and medical condition) and outcome measures. Data were compiled and structured based on the primary goals of this scoping review. The research centered on investigating the holistic influence of rebounding exercises within the realm of rehabilitation. Its primary objective was to analyze the broad-ranging effects of these exercises on the rehabilitation process and various health parameters. By exploring the comprehensive impact of rebounding exercises, the study aimed to ascertain their effectiveness in facilitating physical and functional recovery, addressing specific rehabilitation objectives, and promoting overall health. The quality assessment was carried out using suitable tools corresponding to the research design. The Cochrane Collaboration's risk assessment tool was applied for randomized controlled trials. Two reviewers evaluated the quality, and any discrepancies were resolved through discussion.

Data analysis

**Figure 1:**
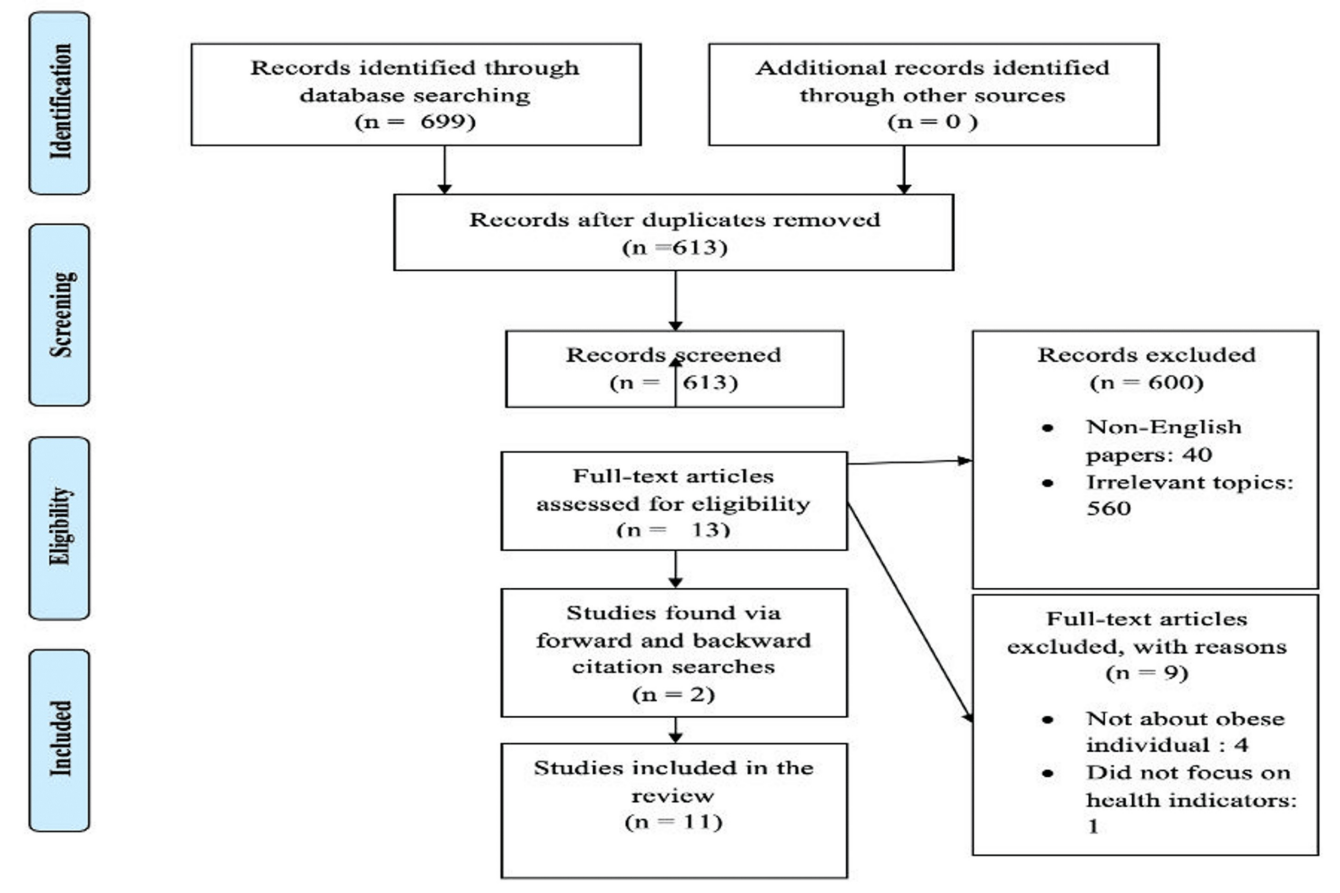
Flowchart of the procedure

A standard method was used to summarize and present the results of the included studies, considering the exploratory nature of this review. The findings were analyzed based on the identified themes, and their implications for the use of mini-trampoline exercises in rehabilitation were examined. The flowchart of the procedure is mentioned in Figure 1.

An overview

Body Composition

The scoping review revealed that participation in mini-trampoline exercises led to a significant decrease in body composition compared to the control group, irrespective of the duration of the training session. Similarly, a study conducted in 2021 observed significant improvements in body fat percentage and bone density in female participants who exercised three times weekly for 12 weeks [10]. A study was carried out where researchers noted a notable enhancement in anthropometric measurements and body composition, encompassing measurements such as circumferences, fat mass, lean mass, and muscular mass [11]. Also, a study concluded that rebounding exercises and diet for four weeks showed a significant difference between the groups in terms of body mass index (BMI) and waist-hip ratio [3]. A study carried out a body jump program three times per week had a positive effect on physical fitness and body composition [12]. A study was undertaken to determine whether mini-trampoline training yielded better results compared to running in terms of body weight and body fat in young men. The study concluded that mini-trampoline exercise could lead to a decrease in body fat percentage [13]. Researchers conducted a study to investigate whether rebound training alters body composition in adult women. The study results indicate that both water-based and land-based rebound training are effective in enhancing body composition [14].

Insulin Resistance

Researchers conducted a study to examine how mini-trampoline exercise programs affect insulin resistance, lipid profile, and central obesity in individuals with type 2 diabetes. Participants followed a 30-minute exercise program three times a week for 12 weeks. Compared to the control group, significant enhancements were observed in insulin resistance, lipid profile, and waist circumference [15].

Physiological Marker

In 2020, a study was conducted to investigate the impact of high-intensity, short-duration exercises on human physiology over an 8-week intervention period. The study results indicated a notable alteration in blood cholesterol levels relative to the volume of oxygen (VO2) and caloric expenditure [6]. Another study in 2021 concluded that incorporating rebounding exercises alongside a dietary regimen over four weeks resulted in a significant discrepancy between the groups regarding the enhancement of lung functions. This improvement was measured by forced expiratory volume in one second and forced vital capacity. The study also noted improvements in BMI and waist-hip ratio [3]. A study was undertaken to determine if mini-trampoline training surpassed running in its effectiveness on body weight and body fat in young men. It revealed a notable contrast in VO2 max between the trampoline group and the running group [13]. Similarly, the research conducted in 2023 explored the impact of mini-trampoline exercise on the physiological parameters of male gymnasts, concluding that the mini-trampoline exercise regimen notably enhanced the anaerobic performance of the experimental group [16]. A study was conducted to investigate if rebound training affects bone health indicators in adult women. The results indicated a decrease in alkaline phosphatase (ALP) and an increase in bone ALP, acid phosphatase, and tartrate-resistant acid phosphatase for both groups. Additionally, femur neck bone mineral density improved in both groups, while lumbar bone mineral density increased specifically in group 2 (land group) following 16 weeks of training twice a week [14]. In 2023, a study concluded that significant reductions were noted in both systolic and diastolic blood pressure values, alongside improvements in lipid and glucose profiles. Furthermore, an increase in work capacity and VO2 max was observed [11].

Strength and Endurance Performance

Witassek et al. conducted a study to assess the impact of eight weeks of standardized jumping training on a mini-trampoline, with three sessions per week. The study revealed a noteworthy rise in running speed at a blood lactate level of 4 mmol and in isometric maximum strength in trunk extension within the intervention group compared to the control group [17]. Another study investigated the impact of trampoline versus resistance training on knee muscle strength and balance in young adults. The findings indicated significant enhancements in knee extension torque, knee flexion torque, and dynamic balance. Consequently, the study concluded that trampoline training can be equally effective as resistance training in enhancing knee muscle strength and dynamic balance in both young men and women [18]. Sellés-Pérez et al. performed a body jump program thrice weekly and showed a significant increase in muscle strength (one-repetition maximum [RM] test) [12]. Malysz et al. investigated whether rebound training affects bone health indicators in adult women. The study findings revealed a notable improvement in muscular strength as assessed by the 1-RM and 20-RM tests in both the rebound training on land and rebound training in the water groups, following a 16-week training program conducted twice a week [14].

Vertical Jump Performance

In a study led by Sellés-Pérez et al., participants engaged in the body jump program three times weekly, resulting in a notable elevation in countermovement jump flight height, squat jump flight height, and 1-RM value during the half squat exercise within the body jump group compared to the control group [12]. Also, a study was conducted to find whether mini-trampoline training was more effective than running in terms of vertical jumps in young women. The study revealed a significant difference in jump height in the mini-trampoline group compared to the running group [13].

Quality of Life

A study led by Cugusi et al. investigated the impact of a mini-trampoline rebound exercise program on the quality of life in overweight women. The findings revealed positive alterations in four out of eight items, along with the mental component summary of the 36-Item Short Form Health Survey. Additionally, according to the Short Form Brief Pain Inventory, there was a reduction in both pain severity and pain interference scores. The study also observed significant enhancements in anthropometric measurements and body composition, encompassing circumferences, fat mass, lean mass, and muscular mass [11].

Challenges and limitations

Utilizing mini-trampoline exercises in rehabilitation holds significant potential, yet it faces inherent challenges and limitations. It is imperative to recognize and address these issues to optimize the effectiveness of mini-trampoline interventions and facilitate their ongoing development. Below, we outline some of the primary obstacles and constraints in this area.

Safety Concerns

Mini-trampolines pose risks of falls and injuries, particularly when users lose balance or attempt advanced maneuvers. For example, landing improperly after a jump may result in ankle sprains or even fractures.

Constrained Fitness Repertoire

Compared to traditional gym equipment, mini-trampolines offer fewer exercise options, which may lead to boredom or difficulty in creating a well-rounded workout routine. For instance, while they excel in cardiovascular exercises like jumping, they may lack versatility for strength training exercises like weightlifting. Mini-trampolines require sufficient space for safe usage, which may be challenging in small living environments or crowded fitness facilities.

Lack of Proper Supervision

Participants may perform exercises incorrectly on a mini-trampoline without proper instruction and supervision, increasing the risk of injury. For instance, beginners may not know how to land properly or perform appropriate warm-up exercises before jumping, leading to strains or muscle pulls.

Durability and Maintenance

Mini-trampoline may have durability issues, especially with frequent use, leading to wear and tear or malfunction. For example, springs may weaken over time, increasing the risk of accidents during exercise sessions. Addressing these challenges through proper safety protocols, exercise guidance, equipment maintenance, and adaptation of exercises can help mitigate risks and maximize the benefits of using the mini-trampoline for exercise.

Future directions

Evidence-Based Protocols

This includes developing standardized and evidence-based rehabilitation protocols specifically tailored to mini-trampoline use for various conditions and injuries, ensuring safety and effectiveness in clinical practice.

Technology Integration

Technology integration includes integrating wearable sensors and digital technologies to monitor and assess patients' movements and progress during mini-trampoline rehabilitation sessions, allowing for personalized feedback and remote monitoring by healthcare providers; and incorporating virtual reality and gamification elements into mini-trampoline rehabilitation programs to enhance engagement, motivation, and adherence to treatment protocols, especially among pediatric and adolescent populations.

Musculoskeletal and Neurological Corrections

Musculoskeletal and neurological corrections include exploring the efficacy of mini-trampoline rehabilitation for a wider range of musculoskeletal conditions beyond traditional applications, such as osteoarthritis, osteoporosis, and chronic pain management; and investigating the potential benefits of mini-trampoline exercises for neurological rehabilitation, including balance training, gait improvement, and functional recovery in individuals with conditions such as stroke, multiple sclerosis, or Parkinson's disease.

Preventive Health and Wellness

This includes promoting the use of mini-trampolines as a preventive measure for maintaining musculoskeletal health, improving balance, coordination, and overall fitness levels across different age groups and populations; and fostering collaboration between physiotherapists, biomechanists, engineers, and other healthcare professionals to advance our understanding of the biomechanical principles underlying mini-trampoline rehabilitation and optimize its therapeutic benefits.

Long-Term Outcomes

The long-term outcomes are to conduct longitudinal studies to evaluate the long-term efficacy and sustainability of mini-trampoline rehabilitation interventions in improving functional outcomes, reducing injury recurrence, and enhancing the quality of life for patients over time. By focusing on these areas of research and innovation, mini-trampoline rehabilitation has the potential to emerge as a valuable adjunctive therapy in the field of physiotherapy, offering safe, effective, and engaging rehabilitation options for a wide range of patients and conditions.

## Conclusions

In conclusion, this scoping review has provided a comprehensive overview of the application, challenges, and future directions of rebound exercises in rehabilitation and fitness settings. The literature highlights the diverse range of applications of rebound exercises, including musculoskeletal rehabilitation, cardiovascular conditioning, balance training, and neurological rehabilitation. However, challenges such as limited research evidence, variability in equipment quality, and safety concerns have been identified. Moving forward, it is essential to address these challenges by conducting further research to establish evidence-based guidelines for the use of rebound exercises in different clinical populations and settings. Additionally, efforts should be made to improve the design and safety features of rebound equipment and develop standardized protocols for rehabilitation and fitness programs. Collaboration between researchers, clinicians, and equipment manufacturers will be crucial in advancing the field and maximizing the potential benefits of rebound exercises for improving health outcomes and quality of life. By addressing these challenges and embracing future opportunities for innovation and collaboration, rebound exercises have the potential to emerge as a valuable and widely utilized modality in rehabilitation and fitness practice.
